# Postsecondary Student Engagement With a Mental Health App and Online Platform (Thought Spot): Qualitative Study of User Experience

**DOI:** 10.2196/23447

**Published:** 2021-04-02

**Authors:** Howard W Wong, Brian Lo, Jenny Shi, Elisa Hollenberg, Alexxa Abi-Jaoude, Andrew Johnson, Gloria Chaim, Kristin Cleverley, Joanna Henderson, Andrea Levinson, Janine Robb, Aristotle Voineskos, David Wiljer

**Affiliations:** 1 Office of Education Centre for Addiction and Mental Health Toronto, ON Canada; 2 Institute of Health Policy, Management and Evaluation, University of Toronto Toronto, ON Canada; 3 UHN Digital University Health Network Toronto, ON Canada; 4 Department of Psychiatry Faculty of Medicine University of Toronto Toronto, ON Canada; 5 Margaret and Wallace McCain Centre for Child, Youth & Family Mental Health Centre for Addiction and Mental Health Toronto, ON Canada; 6 Cundill Centre for Child and Youth Depression Centre for Addiction and Mental Health Toronto, ON Canada; 7 Lawrence S. Bloomberg Faculty of Nursing University of Toronto Toronto, ON Canada; 8 Health and Wellness University of Toronto Toronto, ON Canada; 9 Slaight Family Centre for Youth in Transition Centre for Addiction and Mental Health Toronto, ON Canada

**Keywords:** transition-aged youth, qualitative study, user experience, help-seeking, mental health, postsecondary, mobile apps, adolescent

## Abstract

**Background:**

There is growing interest in using mobile apps and online tools to support postsecondary student mental health, but most of these solutions have suboptimal user engagement in real-world settings. Poor engagement can limit long-term effectiveness and usefulness of these tools. Previous literature has proposed several theories that link factors such as low usability and poor user-centered design to app disengagement. However, few studies provide direct evidence showing what factors contribute to suboptimal user engagement in the context of mobile mental health apps for postsecondary students.

**Objective:**

This study focuses on understanding postsecondary students’ attitudes and behaviors when using Thought Spot, a co-designed mental health app and online platform, to understand factors related to engagement and user experience.

**Methods:**

Students who were given access to Thought Spot for 6 months during a randomized trial of the intervention were invited to participate in one-on-one semistructured interviews. The interviews explored participants’ overall experiences and perceptions of the app, along with factors that affected their usage of various features. All interviews were recorded, and template analysis was used to analyze transcripts.

**Results:**

User satisfaction was mixed among users of Thought Spot. The degree of engagement with the app appeared to be affected by factors that can be grouped into 5 themes: (1) Students valued detailed, inclusive, and relevant content; (2) Technical glitches and a lack of integration with other apps affected the overall user experience and satisfaction with the app; (3) Using the app to support peers or family can increase engagement; (4) Crowdsourced information from peers about mental health resources drove user engagement, but was difficult to obtain; and (5) Users often turned to the app when they had an immediate need for mental health information, rather than using it to track mental health information over time.

**Conclusions:**

Content, user experience, user-centeredness, and peer support are important determinants of user engagement with mobile mental health apps among postsecondary students. In this study, participants disengaged when the app did not meet their expectations on these determinants. Future studies on user engagement should further explore the effectiveness of different features and the relative importance of various criteria for high-quality apps. Further focus on these issues may inform the creation of interventions that increase student engagement and align with their mental health needs.

**Trial Registration:**

ClinicalTrials.gov NCT03412461; https://clinicaltrials.gov/ct2/show/NCT03412461

**International Registered Report Identifier (IRRID):**

RR2-10.2196/resprot.6446

## Introduction

Facilitating access to mental health support for postsecondary students (youth aged 17-29) is critical to preventing underlying conditions from worsening as these transition-aged youth enter adulthood [1-3]. With widespread ownership of cell phones and a willingness to try web-based services, online and mobile mental health apps have been touted as a promising way to provide mental health information and services to this population [4-7]. Delivering mental health support through mobile apps offers several advantages over traditional in-person services, such as improved ease of access, convenience, lower costs, reduced stigma, and user customization [8,9]. Many digital mental health interventions have been developed and offer a wide range of functions, including self-help, symptom monitoring, cognitive behavioral therapy, and psychoeducation for postsecondary students [8,10,11].

Engagement is often considered when understanding the efficacy of digital behavior change interventions, a category that many digital mental health apps fall under. In the literature, engagement refers to both the subjective experience and behavior when interacting with a digital behavior change intervention [12]. According to traditional computer science and human–computer interface research, engagement as a subjective experience can be characterized as feeling focused, attentive, and satisfied when using a digital technology [12]. In comparison, engagement as a behavior commonly refers to the patterns of usage (eg, frequency, duration, retention rate) and depth of usage (eg, use of a specific app feature) [12-14].

Unfortunately, many mobile mental health interventions that target this population face low user engagement [8,13,15,16]. For example, one study found that among 93 mental health apps on the Google Play store, the median 15-day retention rate was 3.9% [13]. Overall, low engagement can hinder an app’s ability to deliver positive outcomes to its users and makes it difficult for researchers to understand the app’s long-term efficacy [8,12,13,15,17,18].

Currently, there is a lack of direct evidence to explain low engagement in the postsecondary student population, but a few theories do exist [17,19]. Some theories posit that personal attitudes toward technology and perceptions about seeking help can lead to disengagement. The design, content, and usability of an app have also been proposed as important factors that affect engagement [17,19]. Exploring these theories will provide stakeholders, such as app developers, clinicians, postsecondary institutions, and policymakers, with evidence about the factors that influence student engagement with mobile mental health apps. These investigations can reveal opportunities and drive strategies for increasing student engagement with mobile mental health apps in the future.

This qualitative study seeks to understand user engagement by exploring postsecondary students’ experiences on Thought Spot, a mobile mental health mobile app. Thought Spot was created through participatory design research methods and usability testing with college and university students [20,21]. Drawing on the social cognitive theory [22] and the theory of help-seeking [23], the intended purpose of this app is to be an online and mobile resource with features that help students find mental health support, build self-efficacy for seeking help, and increase help-seeking behavior [20,24-26]. For example, Thought Spot contains curated information about mental health and wellness resources for youth, and allows them to geo-locate mental health services. The app also displays mental health services and resources that do not require in-person visits, such as websites, apps, and phone or chat support lines tailored to youth. An in-app crowdsourcing function enables users to add mental health and wellness resources, rate them on a 5-star scale, and write reviews for others to see. Newly added resources appear on a timeline that is updated in real time. Resources are categorized using tagged keywords to allow for easy and intuitive searches. A mood-tracking journal enables users to privately record their thoughts and moods during their help-seeking process and to geo-locate where these thoughts occurred.

The primary objective of this qualitative study is to identify factors that affect postsecondary students’ attitudes and behaviors when using the Thought Spot app, and to describe how those factors affected user experience and subjective user engagement.

## Methods

### Study Design

This qualitative study is part of a larger study that includes a randomized controlled trial (RCT) to evaluate Thought Spot [20,27]. The qualitative approach serves to explore questions that complement the larger study, including those that examine factors affecting adoption of and user engagement with Thought Spot for seeking mental health support. It describes the who, what, and where of participant experiences [28]. This study is also a continuation of the participatory design research process and a progression from prior work on Thought Spot [20,24,25]. In contrast to previous qualitative evaluations of Thought Spot in theoretical situations, which were emulated during co-design workshops and usability tests, this study focuses on the experiences of youth participants who used the app in their day-to-day lives during the RCT [20,24,25,27].

### Recruitment, Sampling, and Participants

Participants were recruited to the qualitative phase of the study through purposeful sampling of a subset of students from the intervention group who completed the Thought Spot RCT [27]. Students who indicated in an end-of-study usability survey that they were interested in participating were identified as potential participants for the interviews. The survey explained that the study would involve a 30- to 60-minute in-person or telephone interview about the user’s experience with Thought Spot, and that compensation would be provided. A purposive sampling strategy determined who would be selected for an interview. During the RCT, intervention group participants were sent an email on how to download and access Thought Spot on their personal device [20]. Participants were asked to use the app as needed and they were free to use it in whatever manner they liked. As such, participants were only selected to participate in qualitative interviews if they had logged into the Thought Spot app more than once during the trial between March 2018 and June 2019. User activity was verified by one member of the research team (JS) through a filtered search of Thought Spot’s user activity data logs. Second, participants were purposefully sampled in 2 groups because the RCT was a longitudinal study and participants who received Thought Spot started the study at different times. In January 2019, changes were made to Thought Spot to address technical issues, resulting in small differences between the versions used by study participants. Consequently, to obtain a comprehensive picture of user experience and user engagement, participants were grouped into those who finished the Thought Spot trial before January 2019 (Group A) and those who were participating in the trial after that date (Group B). The research team also purposefully sampled a 50:50 split of users with high usage/satisfaction and low usage/dissatisfaction in both groups. Usage and satisfaction were determined from separate analyses of individual-level usage data from the app and participant scores on the adapted Usefulness, Satisfaction, and Ease of Use (USE) questionnaire, respectively [29] (Shi et al, unpublished data, 2021). The adapted questionnaire was part of the end-of-study usability survey administered to all participants in the intervention arm to evaluate usefulness, ease of use, ease of learning, and satisfaction with the Thought Spot app [20,29] (Shi et al, unpublished data, 2021). The full reporting and analysis of usage data and USE questionnaire responses are reported elsewhere (Shi et al, unpublished data, 2021). During the purposeful sampling process of this study, if there were no more participants with high usage/satisfaction or low usage/dissatisfaction left to sample, the research team sampled participants with mixed USE questionnaire scores/usage, such as those with high USE score and low usage or low USE score and high usage. Applying findings from the separate analyses of USE questionnaire responses and usage data, the research team used the median USE Questionnaire Score, 53.78 (IQR 38.89-67.78) out of total score 100, to discern high/low satisfaction users (Shi et al, unpublished data, 2021). The median number of clicks, 14 clicks (IQR 6-22), was used to discern high/low usage users (Shi et al, unpublished data, 2021). Overall, the purposeful sampling criteria were intended to identify a sample of participants with varying degrees of usage and different perceptions about Thought Spot’s usability.

Students were invited to participate in an interview through an email that contained a summary of the qualitative study and an informed consent form [14]. Participants were offered an honorarium of CAD $40 (USD $32). Interested individuals submitted a signed consent form and arranged a phone or in-person interview with the research analyst and research trainee (BL and HW, respectively).

The study was approved by the Research Ethics Boards at the Centre for Addiction and Mental Health (REB #023/2017), George Brown College (REB #6004416), Ryerson University (REB # 2017-196-1), and the University of Toronto (REB# 00034725). The study was conducted between February 2019 and August 2019.

### Data Collection

After obtaining informed written consent from participants, 2 members of the research team (BL and HW) conducted semistructured interviews using the question guide presented in Multimedia Appendix 1. The interviewers have formal education in health informatics and received training and support from other members of the research team who have expertise in qualitative research. The domains covered in the question guide included general impressions of Thought Spot and its features, the utility and impact of the app for help-seeking and finding resources, how and why the app was used, areas of strength and weaknesses, how using the app related to other help-seeking experiences, and suggestions for how to improve the app. The interviewers used a “funneling” approach for the interview guide [30]. First, they invited participants to share their experience and perspectives on Thought Spot and how they used it to meet their needs. Based on the response, the interviewers probed specific topics, such as usage patterns, changes to the help-seeking process, and what the participant liked or disliked about the app. The interview guide was adjusted iteratively when new patterns emerged during the interviews. Interviews were conducted until the researchers felt that further data collection did not add more depth to the emergent codes or themes [31].

All interviews were audio-recorded, deidentified, and transcribed verbatim by a professional third-party service. Members of the research team checked the transcripts for accuracy and corrected discrepancies.

### Data Analysis

The research team used a comparative and iterative thematic approach to developing themes from the interview transcripts. Two authors (HW and BL) analyzed the transcripts to explore themes related to on-app user behavior, motivation for usage, perceptions of the app, and suggested improvements. The analysis followed the procedural steps recommended by Brooks et al [32] for template analysis because this method permits the inclusion of predefined codes. The research team applied steps from directed content analysis to include preliminary codes from human factors research into the analysis, such as appearance, layout, navigation, and ease of use [33,34].

HW and BL reviewed an initial subset of 3 randomly selected interviews to familiarize themselves with the data before coding. Preliminary coding was then completed on the subset, where keywords were highlighted to guide the development of an initial coding template. Highlighted text from the 3 transcripts was clustered into meaningful codes. Related codes were clustered into a hierarchical structure, with narrow codes organized as subcodes for broader themes. HW and BL created a definition for each theme in the initial coding template and presented them to the rest of the research team, along with exemplary quotes. The research team reviewed the initial coding template and modified it through consensus to ensure representativeness and relevance. The remaining transcripts were coded using the revised coding template. The coding template was revised iteratively, which involved integrating new themes and re-defining existing ones as more transcripts were coded. During coding, members of the research team ensured that all codes relevant to the research question were accounted for in the template. The template was presented to the entire research team at a second session to review and finalize ideas, new themes, and interpretations. Having the broader research team review the template ensured that all perspectives were incorporated in the analysis.

## Results

### Demographics of Participants

Baseline characteristics of the participants are presented in Table 1. The research team was satisfied that they were not seeing any new data after 17 interviews. A total of 11 interviews were conducted via telephone calls and 6 were conducted in-person; 9 students from Group 1 were interviewed in March 2019 and 8 students from Group 2 were interviewed in June 2019. A total of 13 female (76%) and 4 male (24%) students were interviewed, which reflected the demographics of our RCT study of 481 participants (190/241, 78.8%, identified as female). All 3 academic institutions from the RCT were represented.

Following our template analysis, 5 main themes emerged based on users’ experiences and perceptions of the app: (1) Students valued detailed, inclusive, and relevant content on the app; (2) Technical glitches and a lack of integration with other apps affected overall user experience and satisfaction with the app; (3) An app’s features can extend beyond the users to support their peers; (4) Crowdsourced information from peers about mental health resources was valuable and sought after, but difficult to obtain; and (5) Users often used the app when they had an immediate need for mental health information, rather than using it to track mental health information over time.

**Table 1:** Baseline characteristics of participants who were interviewed (N=17).

Characteristic		Value
**Gender, n (%)**		
	Female	13 (76)
	Male	4 (24)
	Other	0 (0)
Age (years), median (IQR)		23.1 (20.9-25.6)
Interview time (minutes), median (IQR)		32.4 (30.9-34.6)
**Classification of participants (USE^a^ questionnaire rank and usage data rank), n (%)**		
	Low satisfaction^b^ and low usage^c^	3 (18)
	Low satisfaction and high usage	7 (41)
	High satisfaction and low usage	3 (18)
	High satisfaction and high usage	4 (24)

^a^USE: Usefulness, Satisfaction, and Ease of Use.

^b^Low satisfaction is defined as users with a ≤50th percentile USE score.

^c^Low usage is defined as users with ≤50th percentile number of clicks.

### Students Value Detailed, Inclusive, and Relevant Content

Many students believed that getting information about mental health resources was valuable in both the short and long term. Many appeared to use Thought Spot as an information-gathering tool to learn about nearby mental health services. Opinions about the app content appeared to affect user satisfaction. For example, some users were pleased with the level of detail and diversity of mental health information that the app provided (all quotes are presented verbatim, but to improve readability, some filler words such as *like* and *um* have been removed):

I liked, when you clicked it, it would tell you what kinds of services it offered. ...There were actually details. ...It showed me what the services were. It showed me what the hours were. It showed me the address. And that's all I really needed at the time. Maybe if I had investigated further, I would have realized that I needed other things, but no. It was good. I was satisfied with it.

P14

By contrast, other users were dissatisfied with the content. For example, a few commented on the lack of breadth, depth, and relevant information. These users wanted more services to be available on the app and sought additional details about them.

I just have utilized a lot of services...so when I was going in just kind of looking, a bunch of things just weren’t included or listed, so I was like, I’m not really sure what people are going to be finding when they come in here...I think that was the only drawback to it, or I guess the piece that it didn’t meet was having all of the relevant services listed.

P08

Some users were curious about the kinds of support provided by a mental health service, such as whether it offered peer support, professional counseling, or another form of support. There were also suggestions to include more background information about the people providing these services, because it helped users gauge whether a service was inclusive of their needs and preferences.

Everybody knows you can go to counselling in your school...but someone who is of colour like might not want to go into that space...so there’s a space that’s hosted by the student’s union that gives peer support but it’s not called peer support...having that added into the app could be really good. I found it really helpful and it’s very inclusive, that might be a better way for students.

P07

There were also other comments related to inclusion, as several users said that the content on the app was too general and did not account for their unique circumstances. For example, some users said that the information on the app did not fully consider factors such as where they lived, where they went to school, whether they had health insurance, and how long it would take to access a service. Consequently, it made it challenging for some participants to understand whether a services or resource was relevant for them.

...it didn’t really take into account the resources I had as a student...it kind of treated all situations as equals, so let’s say, I do have insurance coverage...I know it’s better for me to go in to somebody I can pay for and that can see me sooner, but it’s not necessarily that the app took that into consideration.

P02

I go to [School A] and I found a lot of the places were close to [School B] or close to [School C] I just remember that I couldn't use the full app, because I don't live in Toronto and I don’t have a lot of time when I'm down there.

P16

...if I'm like [School C] student...am I really not allowed to use the [other] school's app...it doesn't really divide or split between [the different types of] professionals and students...So some people may think, “Oh, this doesn't apply to me”....

P13

Other participants wanted to access more content, tools, and strategies to help them manage their mental health concerns, rather than simply being directed to mental health services or resources. Some wanted more support to be delivered directly through the app.

...I have anxiety, so I try to use apps like Calm or different ones that help me calm my anxiety, like Breath, all those different apps...I thought maybe this app was going to help me get those kind of features that the other apps have, like strategies to deal with my mental health issues like anxiety and anxiousness and stress and daily stress, but really it was just providing me with different places to go, I believe, if I understood the purpose of the app properly. So the app wasn’t giving me tools, it was just redirecting me.

P05

Participants indicated that it was important for content to be comprehensive and relevant. They were satisfied when mental health information details were relevant to their circumstances, needs, and preferences. The lack of relevant details also made it difficult for some users to assess whether content on the app was applicable to them and may have decreased their willingness to use Thought Spot. Furthermore, participants indicated that the app provided sufficient information to get a preliminary and surface-level understanding of what mental health services or resources were offered, but that it lacked the level of detail that some participants needed to motivate them to try the resources or services.

### Technical Glitches and a Lack of Integration With Other Apps Affected Overall User Experience and Satisfaction With the App

Several participants identified technical issues as a source of frustration and inconvenience when navigating the app. They described occasional glitches and system lag when using the search and filter feature. This feature was designed to help students make custom searches by selecting key terms to narrow down the services and wellness locations most applicable to them. However, technical issues prolonged the amount of time it took some users to find and retrieve information:

...I tried the search. I tried to look at different features that it had...in the first few times I tried it, it was kind of glitchy and I had to go back and restart.

P05

Sometimes when I went to do something it takes a couple of tries to get the map moving, or if I want to search something, it does take a couple of tries to get it to work, but it doesn’t happen all of the time.

P03

Integration between Thought Spot and other apps was discussed by several participants. They reported using the app alongside other tools, such as Google and journaling and wellness apps, when looking for mental health resources. However, some wanted an all-inclusive app that could connect them to a variety of these tools directly through the app. The current version of Thought Spot does not integrate with other apps, and some students expressed dissatisfaction with the cumbersome process of switching between several platforms. Some felt that improved integration could create a more seamless experience and increase the likelihood that they would take action after accessing information on Thought Spot:

I like to have everything sort of integrated into one application. So given the option, like if I was tracking my fitness app—I once did have a calorie counter and a meal tracker and my monthly menstrual cycles all in the same app, just because it’s too much work to have to go and change apps, and you know, it’s more work and I’d be less likely to do it.

P09

Despite technical issues and lack of integration, some participants were still intrigued by Thought Spot’s potential usefulness for students:

...I remember having some trouble with how it operated it on my phone, and that prevented me from using it a lot, I think, during the study, but also, I thought it was a really great idea.

P15

Specifically, a few participants liked the app’s goal of helping students access mental health support and felt that updating and optimizing the app further would resolve issues related to user experience:

...so maybe in the future we have more financial resource to support this application, I think it should be better, and in the long term I think it's really good for students.

P12

Participants saw value in the app itself, but the quality of Thought Spot’s user experience varied, with some users encountering more issues than others. The main concerns about slow loading and integration with other apps may have interfered with their ability to engage meaningfully and fully with the app.

### The App’s Functionalities Can Extend Beyond the User to Support Peers

Several participants reported using Thought Spot to help their friends and family members. They described sharing resources and services with others who needed mental health support, even when they did not immediately require services or resources themselves:

...I shared the app with my friends, and with some of my friends that have...something they don't want to talk with about to the family or relatives, so I introduced them [to] this app.

P12

I didn’t necessarily go to all of them, but I sent friends to some of the places, like when they needed to go somewhere, I would say, you know, there’s this place, it’s 100 meters away.

P09

The potential of Thought Spot to play a role in providing peer support for mental health concerns was discussed by some participants. They described mental health as a sensitive topic and thought that it was valuable to share information on the app because it might be more trustworthy. One participant suggested adding value by being able to directly share the information with a friend through channels such as social media:

Yeah, refer your friends or share it with your friend, because you know what? With this kind of very sensitive issue, sensitive information, you just believe what you trust or believe. So that's why refer your friend, introducing your friend, that should be a function in the app—to share with your friend.

P12

Even though some participants did not have a pressing need for mental health help themselves, they used the app to become messengers of mental health–related information within their social circles. In situations where a peer required support, participants explained that Thought Spot gave them information that they could share with their peer and that would help them access the resources or services they needed.

### Crowd-Sourced Information From Peers About Mental Health Resources Was a Driver of Engagement, but Was Difficult to Obtain

Thought Spot enables students to crowdsource information, that is, to add mental health services and self-care locations (classified as “spots”) and to provide reviews about these resources. Most users agreed that peer reviews were important and valuable because reviews about mental health resources are often difficult to find and reading about other people’s experiences can increase motivation to access services:

Having the reviews and the comments from peers who have utilized those different groups was...a huge thing that doesn’t exist anywhere.

P08

The same participant added that evaluating the quality of services was challenging or tricky, and seeing diverse peer reviews gave them a more balanced perspective about a “spot” or resource:

And I think through the reviews I’m able to get a little bit more of, kind of a sense of, the vibe and not necessarily the service offered, to know if...I would feel comfortable or okay with it. Yeah. I think that’s a big one, because it’s definitely hard to review any kind of mental health services, especially because people go in in such different places with such different experiences...someone could have a horrible experience just because the person, the professional they were working with or the clinician just was not equipped to deal with that situation, but is amazing for someone else. So I think it’s definitely kind of a balance there.

P08

Although participants valued others’ input, many found it difficult to add resources and post reviews, so they did not use these crowdsourcing features. Participants gave a wide range of reasons they did not contribute. Some attributed their lack of engagement with these features to infrequent usage, lack of motivation, forgetfulness, or insufficient experience with mental health services:

I didn't because I didn't use it for that long...if I was using it for like a more consistent basis, then I would have been able to use it or potentially review any of the spots. Or maybe it's just—sometimes I forget. Honestly, I've not been one to review things a lot...sometimes I'd rather live in the moment than review it. So it could be a really unique aspect of it, if you do have users who are really consistent on reviewing things, but I don't think every user wants to review everything.

P16

The few participants who engaged in crowdsourcing appeared to do so because they were motivated to help fellow students.

When participants were asked to suggest ways to encourage engagement with crowdsourcing, a few acknowledged the complexity and difficulty of motivating others. They were uncertain whether giving incentives or following up with service users would improve participation:

I don’t know what you could use as a motivator...It doesn’t have to be anything of any actual value, but having some sort of—an appearance of a reward at the end of something tends to motivate people, so that might be something that would maybe help.

P09

Peer-contributed information about mental health services and resources appeared to be a driver for user engagement with the app because this kind of information is hard to find elsewhere. Students wanted to hear from their peers to help them evaluate whether a service was the right fit.

### Users Often Used the App in Response to an Immediate Need for Mental Health Information

User engagement appeared to be driven primarily by reactive rather than proactive behavior. Most participants reported using Thought Spot as a tool to learn about mental health resources during times of need. Several participants opened the app only when they were experiencing anxiety, depression, or other symptoms of poor mental health. As one user explained:

Actually, just when I need to, when I have some problem or issue or my friend happen[s] to ask me so I just show it to him....

P12

Some users reported using the app infrequently because they were not experiencing mental health issues during the 6-month study period. However, these users indicated that they would be willing to rely on Thought Spot if problems arose, as one user described:

I didn’t end up going to any of them more than once or twice, even the thought of just having it there, knowing that I could use it if I wanted to, provided a level of comfort that helped when, you know, there were things that would make you spiral or you were not thinking very clearly or very logically.

P09

Although Thought Spot has features that can be used daily, such as adding reviews, crowdsourcing resources, and mood tracking, some users said they seldom engaged with these features.

I didn’t really use the mood tracker...although I’m just bad at tracking things in general, so I guess in that way I could’ve used it regularly, but other than that, nah...because in terms of finding resources, your search kind of stops as soon as you’ve found something that works for you.

P02

While several participants said they did not regularly use Thought Spot to search for and access mental health support, they identified it as an option that they could rely on if they ever needed help.

## Discussion

### Principal Findings

#### Factors That Affected Postsecondary Students’ Engagement With a Mental Health App

This study is among the few that describe factors affecting postsecondary students’ attitudes, behaviors, engagement, and user experience when using a mental health mobile app. Participants identified the comprehensiveness and relevance of app content, user experience, integration with other apps, peer support, and reactive (versus proactive) behavior as factors that affected engagement. To varying degrees, these factors appeared to influence students’ use of the Thought Spot app for mental health help-seeking and their willingness to use it in the future.

How engaged users were on Thought Spot appeared to be affected by factors that can be grouped into 5 themes. First, positive experiences on the app were tied to whether it delivered mental health information that users found to be concise, inclusive, relevant to their needs, and that included meaningful details. Dissatisfaction with the content appeared to decrease users’ willingness to engage with the app and to use it to support their mental health. A second theme related to engagement was user experience and integration with other apps. Despite technical issues and lack of integration, some participants were still willing to engage with Thought Spot if they believed they could benefit from it. Third, some participants were motivated to engage with Thought Spot to support friends and family members who needed mental health support. Accessing crowdsourced information was a fourth theme related to engagement. Some participants used the app to read reviews about their peers’ experiences with mental health resources and services. Fifth, engagement with the app appeared to be driven by reactive rather than proactive behavior, that is, participants often used the app when they had an immediate need for mental health information. These themes provide important insights into factors that affect the engagement of postsecondary students with mobile apps in the context of seeking help for mental health issues.

#### Research Theories on Low Engagement With Mobile Mental Health Interventions

The findings of this study that relate to user engagement are consistent with those of previous research on mobile health and mobile mental health interventions. That research has proposed general theories for low engagement, and this study adds direct evidence to support several concepts related to engagement [8,15,18,19]. For example, studies have theorized that usability issues, not being user-centered, and lacking relevant information about mental health services limited users’ ability to address mental health problems or progress toward their help-seeking and wellness goals [18,19]. The themes developed in this study that relate to Thought Spot’s content and user experience provide some evidence that supports existing theories of low engagement. For example, several users said that experiencing usability issues such as technical glitches or lacking integration hurt their subjective experience with using Thought Spot, which could thereby jeopardize engagement. Similarly, having difficulties navigating the app and finding relevant content could have impaired engagement because several participants reported it as a source of user dissatisfaction.

#### Information Exchange With Peers and Family Members

The findings of this study complement existing research about barriers to and facilitators of mental health help-seeking among youth, specifically the importance of peer and family relationships [35-37]. For example, previous research has found that many young people prefer informal sources of support, often family and friends, when they are seeking help or information about mental health [35-37]. Participants in this study also expressed that preference and indicated that they would use the app to help family or friends access mental health information and services. Some participants described Thought Spot as a source of accessible and accurate information about mental health services and wanted improved ways to share this knowledge with others. Although sharing and communication features are not part of the current app version, participants in the co-design and prototyping phase of the project recommended embedding peer-to-peer communication within the app.

Although sharing and communication features were not available, participants still found a way to exchange mental health information with family and peers. This type of peer-to-peer communication could be seen as an innovative way of working around the app’s limited features. Sharing content from Thought Spot suggested the potential of apps to improve awareness of mental health services and resources, which is the most common knowledge-related barrier to seeking help [35]. Moreover, the findings reiterate the crucial role that peers and family members often play during help-seeking [35-37].

The findings are also consistent with theories that identify peer support as a way to improve user engagement [19]. Several participants described crowdsourcing as an appealing strategy for students. Peer reviews of mental health services were a valuable decision-making aid for some participants who were seeking help for themselves. They explained that these kinds of reviews are scarce, but that they are more relevant and trustworthy than information from an unknown source. The request for more reviews from mental health service users underscores the importance of peer support as a feature that increases user engagement. Overall, the study provides new evidence to support existing theories about low engagement with mobile health and mobile mental health interventions.

#### Challenges of Co-Designing Apps

The Thought Spot project was student led and many postsecondary students were actively involved in deciding what features to include in the app that was evaluated during the RCT. Several findings from the participant interviews echo what students had discussed during the co-design workshops and focus groups in the earlier stages of Thought Spot’s development [25]. At that time, students suggested adding more peer support, including features that would enable communication between users and with social groups. They also requested more information about service costs, accessibility, and languages spoken [25]. The research team considered these suggestions for the optimization phase of the app’s development, but they could not be implemented for various reasons. For example, the databases from which Thought Spot draws information do not collect information on cost of services, accessibility, or wait times. Project cost constraints made it unfeasible to add complex features such as integration with other apps. Direct peer-to-peer interaction features were not implemented because they pose a high risk for misuse and the research team lacked the resources to monitor this activity for safety. Nonetheless, the similarities between students’ perspectives during the development of Thought Spot and after the RCT show that participatory co-design research can be a useful tool for identifying key features that influence user engagement in the final product.

This study is also one of the few studies of mobile mental health interventions that points to the challenges during the co-design process of balancing user needs and perspectives with project resources, feasibility, and risk [38,39]. In future studies, it may be valuable for other researchers to also discuss the consequences when co-design suggestions cannot be implemented. Likewise, it can be useful to learn about the complex decision-making process that developers undergo when choosing what features to include or exclude. Doing so could identify areas of caution and guide other mobile mental health app developers.

#### Comparison With Mobile Mental Health Assessment Frameworks

Mobile mental health assessment frameworks, such as those developed by Chan et al [40], Zelmer et al [41], and Stoyanov et al [42], help researchers evaluate apps and guide developers in building high-quality, safe, and effective tools. These frameworks describe key considerations, including fit to target group, functionality, information quality, integration, user-centeredness, usefulness, usability, security, and transparency [40-42]. Although the frameworks are useful guidelines, it is unclear how much engagement with the technology will change when the framework criteria are satisfied [40-42]. The findings of this study provide preliminary indications, given the similarities between several criteria in the 3 frameworks cited above and the themes developed in this study. For example, functionality and usability criteria, which refer to an app’s performance, reliability, and ease of use, are similar to the themes that emerged in this study that relate to user experience and willingness to use Thought Spot [40-42]. Likewise, framework criteria about fit to target group, information quality, and usefulness are reflected in this study’s theme that links engagement with the provision of app content that is detailed, inclusive, and relevant [40-42]. These complementary findings indicate that content and usability improve engagement, but further investigation is required to measure the impact.

Findings from this study suggest that some participants prioritize usefulness over the user experience. However, the assessment frameworks described above do not rank the importance of each criterion [40-42]. It may be useful for future studies to explore the relative impact that each criterion has on engagement. That knowledge could help app developers determine what features or functions to prioritize to maximize adoption and engagement. Moreover, incorporating this information into existing assessment frameworks could increase their practical value in guiding the development of projects with limited resources and time constraints, such as Thought Spot and many publicly funded co-designed projects [39].

It is important to note that sustained usage is not guaranteed even if all framework criteria are satisfied. For example, in our study, some students used Thought Spot only during times of pressing need, which could result in infrequent and sparse engagement, regardless of the quality or usefulness of the app. This behavior suggests that user engagement is context dependent, and that an app can be useful despite low engagement with it.

### Limitations

This study had several limitations. Because of purposeful sampling, the participants may not be representative of all users in the RCT. In addition, the study did not factor mental illness diagnoses into the recruitment strategy or thematic analysis, which means that we may not have captured the perspectives of students who are in greatest need of mental health support. During the recruitment process, the research team was not able to engage students who did not use the app, so our analysis did not include feedback from the most disengaged and disinterested students. Lastly, the findings may not be fully generalizable to mental health solutions with different functions. For example, Thought Spot functions primarily as a stand-alone app to assist with finding and navigating to mental health resources, but the factors that encourage engagement with it may differ from the factors that encourage engagement with an app that involves direct communication with a mental health professional (eg, cognitive behavioral therapy or counseling apps).

### Conclusions

This study demonstrates that content, usability, user-centeredness, and peer-to-peer communication are determinants of engagement with apps such as Thought Spot among postsecondary students. Failing to meet participants’ expectations on these dimensions led to disengagement with the app. The findings highlight the challenges of balancing user needs and perspectives with project resources, feasibility, and risk during the co-design process, as well as the difficulty in predicting which app features will be successful, even after a thorough co-design process. The findings of this study support criteria for engagement proposed in several mobile mental health assessment frameworks. However, neither this study nor existing theoretical frameworks have determined whether certain criteria have a greater impact on engagement than others. Future studies that measure the relative importance of each criterion for user engagement would yield insights that could help app developers prioritize certain features or functions, creating interventions with greater engagement and that reflect what students want and need when they are seeking mental health support.

## Appendix

Multimedia Appendix 1 Interview guide and probing questions.

## Abbreviations

RCT: randomized controlled trial
USE: Usefulness, Satisfaction, and Ease of Use
