# Supplementary material for: Postsecondary Student Engagement With a Mental Health App and Online Platform (Thought Spot): Qualitative Study of User Experience
Source: JMIR Ment Health. 2021 Apr 2;8(4):e23447. doi: 10.2196/23447 (PMC8052598; doi:10.2196/23447)
Supplement: Multimedia Appendix 1 [file mental_v8i4e23447_app1.docx]

**Multimedia Appendix 1:** Interview guide and probing questions.

| Number | Question and probes |
| --- | --- |
| 1 | What is your impression of Thought Spot?   - What was your experience like? |
| 2 | - Do you think the app met its goal to help students find resources? Why or why not? |
| 3 | Did you find the types of resources that you were seeking?   - If you did not, what kinds of resources were missing or what challenges did you have (e.g. resources that could not be accessed due to technical issues, incorrect/irrelevant information)? |
| 4 | Did the app change your ability to seek support and resources for yourself or others? How?   - Do you feel better able to manage your mental health and wellness using the app? - Can you tell me a bit more about this? |
| 5 | How would you modify the app to improve your experience with navigating the system and to seek help for yourself? |
| 6 | If you were to think about health and wellness services and resources, how did the app change your knowledge or awareness about these services and resources?   - How has it increased your knowledge about health resources? The types of supports available? Mental health in general? - Can you walk me through an example of one of these experiences? |
| 7 | Did you use the app to record your thoughts and moods?   - Yes—Why did you choose to record your thoughts and moods? - No—Why did you choose not to record your thoughts and moods? |
| 8 | Did you add spots, report spots/reviews or add reviews on Thought Spot? Why or why not?   - When users collaborate by adding spots and resources, this is called crowdsourcing. Did you participate in crowdsourcing? Why or why not? - Is crowdsourcing important to you? Why or Why not? |
| 9 | What would encourage you to participate in crowdsourcing (or adding spots and reviews) for an app like this?   - What barriers may prevent you from participating in crowdsourcing? |
| 10 | Can you tell me how this app supports your mental health and wellness (positive, negative or neutral)?   - Do you feel more empowered to find resources that you need by using Thought Spot? - Has there been a change in your level of confidence in interacting with services? |
| 11 | Is there anything more you would like to add or think is important for me to know?   - What else about this app? - What else about this app would you like to change? |
